# Implementing Teledermoscopy to Shorten Doctors’ Diagnostic Process for Suspected Skin Cancer: Observational Pilot Study

**DOI:** 10.2196/66782

**Published:** 2025-05-30

**Authors:** Rebekka Nordahl Larsen, Niels Kvorning Ternov, Kristian Kidholm, Tine Vestergaard

**Affiliations:** 1 Department of Dermatology and Allergy Centre, Odense University Hospital, Kløvervænget 15, Odense, 5000, Denmark, 45 65412705; 2 Department of Plastic and Reconstructive Surgery, Herlev and Gentofte Hospital, University of Copenhagen, Copenhagen, Denmark; 3 Department of Clinical Research, Centre for Innovative Medical Technology, Odense University Hospital, Odense, Denmark

**Keywords:** skin cancers, teledermatology, teledermoscopy, malignant melanoma, nonmelanoma skin cancers, time

## Abstract

**Background:**

Skin cancers are the most frequent types of cancer, and the incidence continues to rise. Teledermoscopy is a promising tool in the diagnostic process of potential skin cancer, and new technologies are constantly being developed. However, little information is available on how teledermoscopy affects physicians’ time consumption.

**Objective:**

This study aimed to investigate whether teledermoscopy can shorten the diagnostic process for physicians examining skin lesions suspected of skin cancer.

**Methods:**

We recorded the time primary care providers, dermatologists, plastic surgeons, and pathologists spent examining lesions suspected of skin cancer, with and without teledermoscopy. Furthermore, we looked at five different diagnostic pathways, which reflected the most common ways through the Danish health care system for patients with suspected skin cancer, to estimate the total amount of time physicians spent examining these lesions with and without teledermoscopy.

**Results:**

A total of 118 time recordings were obtained. With teledermoscopy, the diagnostic process was significantly shortened for dermatologists (*P*=.008) but prolonged for primary care providers (*P*=.03). While the use of teledermoscopy saved time in one of the diagnostic pathways, it increased the time spent in the four others.

**Conclusions:**

Our research suggests that the implementation of teledermoscopy could save time for dermatologists and potentially plastic surgeons and pathologists, provided that a sufficient number of benign skin lesions can be accurately diagnosed and excluded from further examination and treatment. In contrast, the implementation of teledermoscopy might prolong primary care providers’ consultation time.

## Introduction

### Background

Skin cancers can be categorized into melanoma and nonmelanoma skin cancers (NMSCs) and are the most frequent types of cancer in Denmark and globally [1,2]. Recent data have shown that NMSC now results in a higher total number of deaths worldwide compared to malignant melanoma (MM), although the individual mortality risk is still higher for MM [3]. For the past decade, there has been a continuous increase in the number of NMSC and MM cases in Denmark [4]. This places a greater burden on a health care system already under pressure from an ageing population, a shortage of health care workers, and the continuous introduction of new, costly treatments [5,6].

In Denmark, primary care providers (PCPs) serve as gatekeepers for all dermatological concerns including skin cancer suspicions. PCPs encounter a broad variety of skin diseases, and studies have suggested that over half of the potentially malignant skin lesions referred to specialists are later diagnosed as benign [7-9]. This could indicate the need for an additional filter function, such as teledermoscopy, to better use the time and resources of health care workers and patients.

Teledermoscopy is a technology within the field of teledermatology where dermoscopic images of skin lesions can be referred to a dermatologist for evaluation [10]. The overall diagnostic accuracy of teledermatology is high, and results from recent studies indicate that accuracy and sensitivity are improving with the emergence of new technology [11]. Some of the advantages of teledermatology include significantly shortened waiting periods for patients and a reduction in the number of referrals for in-person consultations [11-13]. In addition, studies have found that PCPs, dermatologists, and patients have a general positive approach to the implementation of teledermoscopy [14,15]. Still, a few barriers exist when it comes to the implementation of teledermatology in health care systems. Among other things, physicians have reported concerns about lack of clinical information, technical issues, increased workload, and time consumption [13,16].

Only a few studies have measured the time required for consultations using teledermoscopy or teledermatology, and most only report time spent on completing or evaluating telereferrals as additional data points [12,17-19]. Since the area of teledermoscopy is constantly evolving, we wanted to look into a Danish-developed teledermoscopy platform, in order to see the impact and potential gain of implementation for physicians in terms of time consumption.

### Research Objectives

The overall purpose of this study was to investigate whether teledermoscopy can shorten the diagnostic process for physicians when examining skin lesions suspected of skin cancer.

First, we estimated the amount of time spent investigating these lesions with and without teledermoscopy for PCPs and dermatologists. Then time measurements were performed without teledermoscopy for plastic surgeons and pathologists.

Second, in order to compare the total amount of time physicians spend triaging, diagnosing, and treating skin cancer with and without teledermoscopy, we aimed to construct different diagnostic pathways, reflecting the most common ways through the Danish health care system. Time spent by patients and other staff was not included.

## Methods

### Ethical Considerations

This study did not interfere with the patients’ potential treatment for skin cancer. It was conducted as a quality assurance project, which does not need an ethical review by the regional or national research ethics committee [20]. This study was approved by the Danish Data Protection Agency (reference 23/19285).

### Clinical Locations

Time recordings were carried out at multiple locations in the Region of Southern Denmark and the Capitol Region of Denmark. Locations included 2 different primary care centers, 2 different private practice dermatologists, the department of pathology at Herlev Hospital, and the departments of dermatology, plastic surgery, and pathology at Odense University Hospital (OUH).

### Data Collection

Before data collection, staff members from the primary care centers, dermatologist clinics, and the department of plastic surgery had reviewed their schedules in order to trace patients with potential skin cancer. During the designated data collection period, the investigator (RNL) performed the time recordings on days when these patients were scheduled for examination or removal of the skin lesion. We randomly picked the days for data collection at the department of pathology. The dermatopathologists selected the specimens with potential to be skin cancer.

We intended to collect a minimum of 10 recordings at primary care centers and dermatologists with and without teledermoscopy. Furthermore, we aimed to gather a minimum of 10 recordings at the department of plastic surgery and the department of pathology without teledermoscopy. Data were stored in Microsoft SharePoint.

### Inclusion Criteria

At primary care centers, the eligibility criteria included patients presenting at their PCP with concerns about potential skin cancer, regardless of the PCP’s final assessment as malignant or benign. In dermatologist clinics, the eligibility criteria included patients referred by PCPs for suspected skin cancer. At the department of plastic surgery, the eligibility criteria included patients referred by PCPs or dermatologists for skin tumor excision. At the department of pathology, the eligibility criteria included patients with skin lesions suspected of skin cancer, referred by either PCPs, dermatologists, or plastic surgeons.

### Exclusion Criteria

At primary care centers, the eligibility criteria excluded patients presenting with inflammatory skin diseases or follow-up of previous examined benign lesions. In dermatologist clinics, the eligibility criteria excluded physical referrals of patients with more than one skin tumor. At the department of plastic surgery, the eligibility criteria excluded re-excisions and excisions with skin grafts. At the department of pathology, the eligibility criteria excluded re-excisions and biopsy-verified lesions.

### The Teledermoscopy Platform

In this study, teledermoscopy was performed using the teledermoscopy platform Dermloop (Melatech ApS).

#### Primary Care Providers

PCPs used the Dermloop Capture app (version 2.31; MelaTech IVS on an Apple iPhone 11). Each PCP had a personal account they had to log into upon entering the app. In order to send a telereferral to a dermatologist, the PCP had to state the patient’s social security number, answer a short questionnaire about the skin lesion (skin type, recent changes in appearance, tentative clinical diagnosis, risk factors, etc), and take an overview as well as a dermoscopic photograph of the lesion with an attachable dermoscope (Handyscope, Dermlite).

#### Dermatologists

Dermatologists used Dermloop Desktop (MelaTech) on their computers. To be able to see telereferrals sent by PCPs, dermatologists had to log in to their personal accounts. When logged in to the platform, a list of pending and diagnosed skin lesions appeared on the screen. The dermatologists could then click on the pending skin lesions and examine the image, evaluate the quality of the photograph, assess the difficulty of diagnosing the skin lesions, give a diagnosis, and describe a potential treatment and further plan. After submission, PCPs received an email with the dermatologist´s response.

### Time Recordings

#### Overview of Time Recordings

Time recordings were carried out with a digital stopwatch. The number of skin lesions examined and notes about the circumstances (eg, interruptions) were made to detect potential differences that could have an impact on the time recordings. To make time recordings as uniform as possible, we planned that all recordings would be performed by the same person (RNL). Before this project, physicians had been trained in the use of the teledermoscopy platform as a part of other projects.

#### Primary Care Provider

Time recordings were carried out at consultations between a PCP and a patient.

Recordings without teledermoscopy included obtaining a medical history about the skin lesion, small talk, an objective clinical examination with or without dermoscopy and informing the patient about the tentative diagnosis, and further plan. Recordings with teledermoscopy included the same tasks plus photography using the teledermoscopy equipment. Before data collection, PCPs using the teledermoscopy equipment had sent between 4 and 19 telereferrals.

#### Dermatologist

Time recordings without teledermoscopy were carried out at consultations between a dermatologist and a patient referred from a PCP with suspected skin cancer.

Recordings included obtaining a medical history about the skin lesion, small talk, a clinical examination, including dermoscopy, and informing the patient about the tentative diagnosis and further plan.

Time recordings with teledermoscopy only included dermatologists evaluating telereferrals sent from PCPs on the teledermoscopy platform. Recordings included reading about the skin lesion (potential diagnosis, risk factors, etc), examining the lesion based on an overview image and a dermoscopic image, evaluating the quality of the images (clinical and dermoscopic image), and writing a tentative diagnosis and further plan in the teledermoscopy platform.

#### The Department of Plastic Surgery

The time recordings were carried out in an operating room. The procedure was performed by a plastic surgeon with help from a nurse while the patient was under local anesthesia.

Recordings included obtaining a short medical history about the skin lesion, examining the skin lesion, explaining the procedure, preparing for the procedure (glasses, mask, sterilized gloves, and surgical gown), excision of the skin lesion, stitching, bandaging, and providing the patient with information about precautions and potential side effects of the procedure.

#### The Department of Pathology

The time recordings were carried out during histopathological evaluations of skin lesions by pathologists at the department of pathology.

The 2 departments included in our study used different types of microscopes in their histopathological evaluation. Tissue sections at Herlev Hospital were examined with regular light microscopes, whereas tissue sections at OUH were examined on a diagnostic computer screen. At OUH, all slides with tissue sections are scanned by “whole slide scanners” and obtained in an “image management system.” The pathologist can access high-resolution microscopic images of the scanned tissue sections in the image management system on their diagnostic screen and examine the skin lesion [21].

Recordings included reading about the removed skin lesion in the referral, examining the skin lesion (regular light microscopy or diagnostic screen), and describing findings.

Depending on the difficulty of diagnosing the skin lesions, additional tasks, such as reviewing the patient’s electronic medical record or discussing findings with a colleague, might be included in the recordings.

In some cases, only a part of the histopathological evaluation was measured, either because further immunohistochemical staining was required or because the tissue had already gone through this process. Immunohistochemical staining takes approximately one and a half days, which made it difficult to be at the department for both evaluations.

### Construction of Diagnostic Pathways

In this study setup, where time and data collection resources were limited and skin cancer consultations at PCPs appeared sporadically, it was a major challenge to identify relevant cases and follow them at every step of the diagnostic process. Thus, it was not possible to measure the total time physicians spend examining potential skin cancer for each patient included.

**Figure 1.**
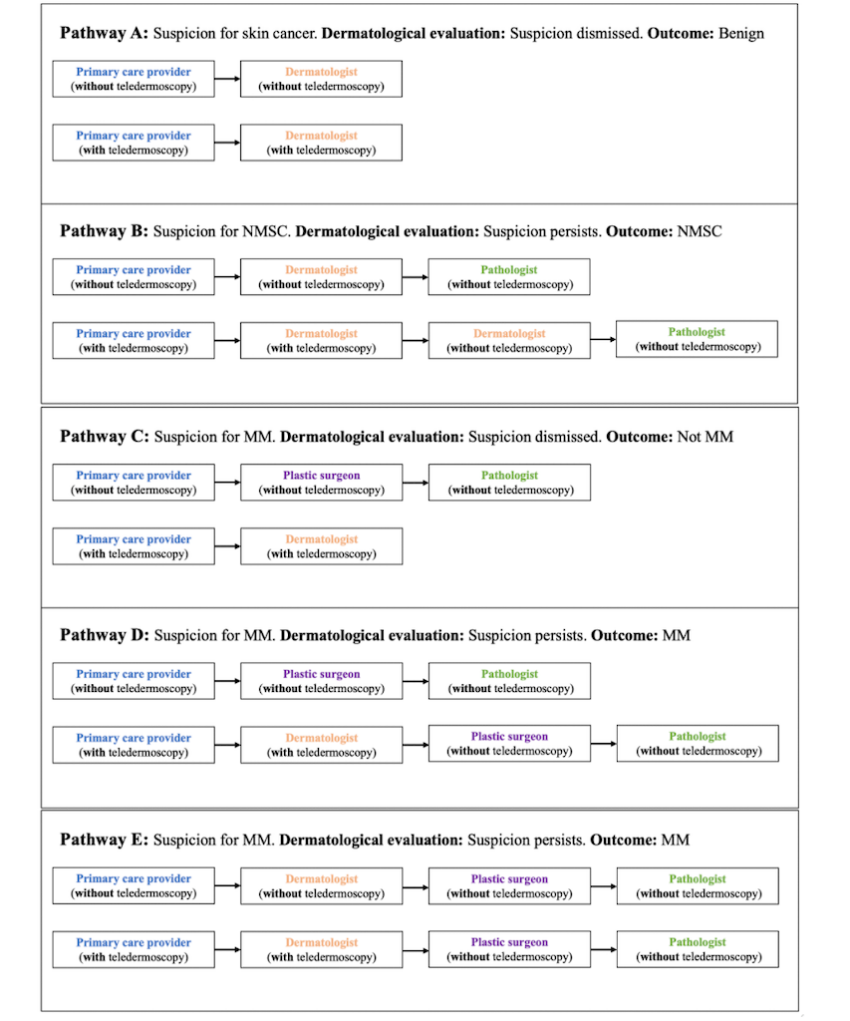
Constructed diagnostic pathways with and without teledermoscopy for patients with suspected skin cancer. Diagnostic pathway A: potential skin cancer. The suspicion is dismissed by the dermatologist, with or without the use of teledermoscopy. Outcome: benign lesion. Diagnostic pathway B: suspected NMSC. Without teledermoscopy: the PCP refers the patient to an in-person consultation with a dermatologist. The dermatologist removes the skin lesion and sends it to a pathologist for evaluation. With teledermoscopy: the suspicion persists, and the patient is referred to an in-person consultation with a dermatologist. The dermatologist removes the skin lesion and sends it to a pathologist for evaluation. Outcome: NMSC. Diagnostic pathway C: suspected MM. Without teledermoscopy: the patient is referred to the department of plastic surgery, where the lesion is removed and sent to a pathologist for evaluation. The pathologist dismisses the suspicion. With teledermoscopy: the suspicion is dismissed by the teledermatologist. Outcome: benign lesion. Diagnostic pathway D: suspected MM. Without teledermoscopy: the patient is referred to the department of plastic surgery, where the lesion is removed and sent to a pathologist for evaluation. The pathologist confirms the suspicion. With teledermoscopy: the suspicion persists, and the patient is referred to the department of plastic surgery, where the lesion is removed and sent to a pathologist for evaluation. The pathologist confirms the suspicion. Outcome: MM. Diagnostic pathway E: suspected MM. Without teledermoscopy: the patient is referred to an in-person consultation at the dermatologist. With teledermoscopy: images of the lesion are sent for evaluation by the teledermatologist. The suspicion persists, and the patient is referred to the department of plastic surgery, where the lesion is removed and sent to a pathologist for evaluation. Outcome: MM. MM: malignant melanoma; NMSC: nonmelanoma skin cancer.

Consequently, we constructed a variety of diagnostic pathways that aimed to reflect real diagnostic processes. For pathways without the use of teledermoscopy, this was done in accordance with guidelines for physicians regarding referral criteria for lesions suspected of skin cancer, published by the Danish Health Authority [22] and the Region of Southern Denmark [23], where so-called cancer patient pathways (CPPs) are described for both MM and NMSC. Pathways with the use of teledermoscopy were constructed based on expertise from a consultant dermatologist (see Figure 1). It must be emphasized that the constructed pathways are simplified and does not cover all possible scenarios. The total amount of time physicians spend examining skin lesions in each of the constructed pathways was calculated by adding together the median amount of time PCPs, dermatologists, plastic surgeons, and pathologists spend on the individual parts of the process.

### Data Analysis

**Figure 2.**
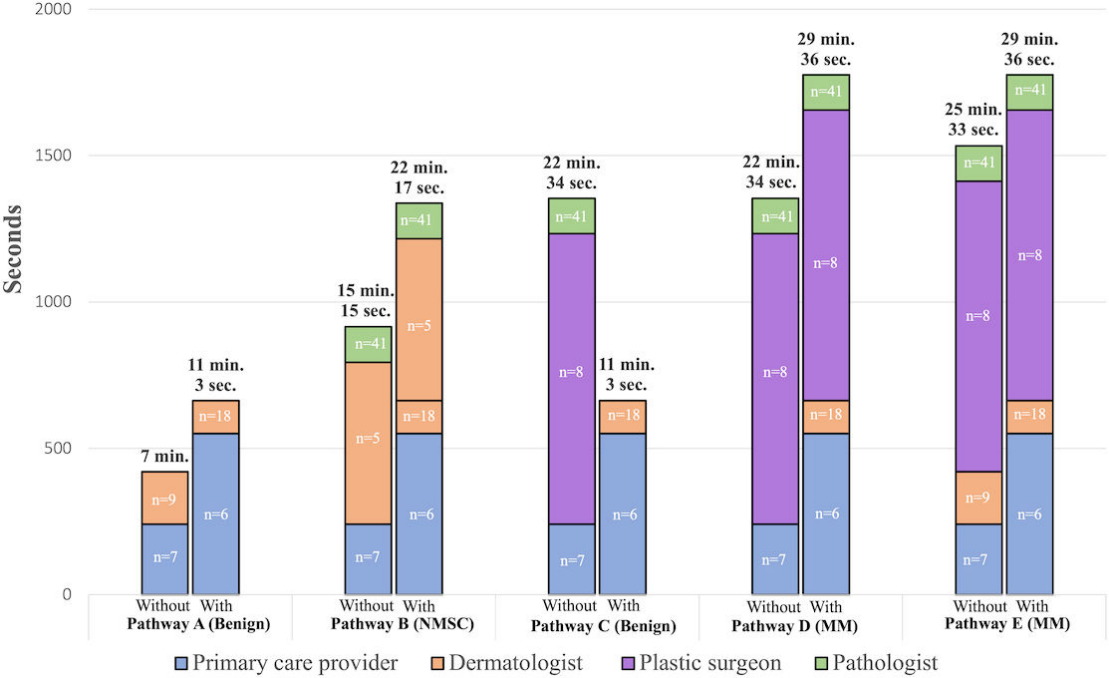
Stacked bar chart showing the amount of time various physicians spend investigating suspected skin cancer for the constructed diagnostic pathways A-E (see legends for Figure 1). The total (median) time physicians spend in each pathway is shown at the top of each column. The height of each segment represents the (median) time physicians in each group spend investigating skin lesions. The diagnostic outcome is shown in brackets. The number of time recordings is shown in each segment. MM: malignant melanoma; NMSC: nonmelanoma skin cancer; min: minutes; sec: seconds; With: with teledermoscopy; Without: without teledermoscopy.

Stata/BE 18.0 was used to calculate all statistical analyses. For each individual part of the process, the Mann-Whitney U test was used to compare time consumption with and without teledermoscopy. *P*<.05 was considered significant. Data are presented as median (IQR). All time statements are presented in minutes (min) and seconds (s), that is, min:s. For each of the constructed diagnostic pathways (see Figure 1), the calculated median for every individual part of the pathway was added together (see Figure 2). The absolute difference between the constructed pathways with and without teledermoscopy was calculated.

## Results

### Baseline Characteristics

Data were collected from April 2023 to December 2023. The dataset comprised 118 time recordings and included 17 physicians examining lesions suspected of skin cancer. When asked if it was acceptable for us to perform time recordings during examinations or treatment for potential skin cancer, no patients declined.

Upon evaluation of the data collected, a total of 24 recordings were excluded due to data not fulfilling the inclusion criteria or to various interruptions or errors during the time measurements. The exact number of time recordings excluded at each department was 5, 3, 8, and 8 at the PCP, dermatologists, department of plastic surgery, and department of pathology, respectively. Thus, a total of 94 time recordings were included in this study.

Tasks included in the procedure at the department of plastic surgery, dermatology, and pathology were repeated per lesion. Consequently, time recordings including more than 1 lesion were divided, resulting in each time recording comprising 1 skin lesion.

In order to make time recordings as uniform as possible, the majority of measurements were carried out by the same person (RNL). Due to time constraints, the physician (TV) at the department of dermatology at OUH made some of the recordings on her own (6.8%), following instructions.

### Individual Parts of the Process

Table 1 shows the data for the individual parts of the diagnostic processes with and without teledermoscopy for physicians. PCPs spend significantly longer time with teledermoscopy, whereas the diagnostic process was significantly shortened for dermatologists. The absolute median differences with and without teledermoscopy was 05:09 for PCPs and 01:06 for dermatologists.

**Table 1.** The amount of time primary care providers, dermatologists, plastic surgeons, and pathologists spend investigating skin lesions suspected of skin cancer with and without teledermoscopy.

Characteristic	Time recordings without teledermoscopy (n=70)	Time recordings with teledermoscopy (n=24)	*P* value
PCP^a^			
Time recordings, n	7	6	—^b^
Patients, n	7	6	—
Skin lesions, n	11	7	—
Number of PCPs involved in the recordings, n	5	5	—
Time, median (IQR)	04:01 (02:41-04:55)^c^	09:10 (08:19-11:59)	.03
Dermatologists (without treatment)			
Time recordings, n	9	18	—
Patients, n	9	18	—
Skin lesions, n	9	18	—
Number of dermatologists involved in the recordings, n	2	2	—
Time, median (IQR)	02:59 (02:05-04:58)	01:53 (01:14-02:56)	.008
Dermatologists (with treatment)			
Time recordings, n	5	—	—
Patients, n	5	—	—
Skin lesions, n	5	—	—
Number of dermatologists involved in the recordings, n	2	—	—
Time, median (IQR)	09:13 (07:13-10:14)	—	—
The department of plastic surgery			
Time recordings, n	8	—	—
Patients, n	8	—	—
Skin lesions, n	8	—	—
Number of plastic surgeons involved in the recordings, n	2	—	—
Time, median (IQR)	16:32 (13:33-18:20)	—	—
The department of pathology			
Time recordings, n	41	—	—
Patients, n	30	—	—
Skin lesions, n	41	—	—
Number of pathologists involved in the recordings, n	4	—	—
Time, median (IQR)	02:01 (01:31-03:11)	—	—

a PCP: primary care provider.

b Not available.

c Presented as min:s, ie, minutes:seconds.

### Constructed Diagnostic Pathways

Figure 2 shows the total amount of time physicians consumed at each of the constructed diagnostic pathways. Teledermoscopy shortened diagnostic pathway C with 11:31, whereas pathways A, B, D, and E were prolonged with 04:03, 07:02, 07:02, and 04:03, respectively.

### Post Hoc Analyses

Despite the use of different types of microscopes at the two departments of pathology involved, no significant difference between the time recordings at Herlev Hospital and OUH was found (*P*=.27).

In Denmark, 400.000 dermatological referrals are made per year, out of which about 30% (120,000) deal with suspicion of skin cancer [24,25]. Assuming 75% (90,000) of these referrals are benign and 25% (30,000) are NMSC [7], the amount of time physicians spend in pathway A and B with teledermoscopy can be estimated as shown in Table 2.

**Table 2.** Calculated total time consumption in hours per year with and without teledermoscopy for primary care providers, dermatologists, plastic surgeons and pathologists, for all constructed pathways.

		Primary care providers (hours per year)	Dermatologists (hours per year)	Plastic surgeons (hours per year)	Pathologists (hours per year)
Pathway A					
	Without TDS**^a^**	6025	4475	—^b^	—
	With TDS	13,750	2825	—	—
	Sum	+7725	−1650	—	—
Pathway B					
	Without TDS	2008	4608	—	1008
	With TDS	4583	5550	—	1008
	Sum	+2575	+942	—	0
Pathway C					
	Without TDS	281	—	1156	141
	With TDS	641	132	—	—
	Sum	+360	+132	−1156	−141
Pathway D					
	Without TDS	203	—	837	102
	With TDS	464	95	837	102
	Sum	+261	+95	0	0
Pathway E					
	Without TDS	203	151	837	102
	With TDS	464	95	837	102
	Sum	+261	−56	0	0
All pathways					
	Total amount of time spend with TDS per year	+10,921	–481 to –632	–1156	–141

a TDS: teledermoscopy.

b Not available.

The most recent report from the Danish Health Authority regarding CPPs for skin lesions suspected of MM stated that in 58% of the cases, the suspicion was dismissed at some point in the diagnostic pathway [9]. The number of patients diagnosed with MM in 2022 was 3038 [4], which corresponds to a total of 7233 CPPs for suspected MM, with 4195 (58%) dismissed cases in 2022 [9]. The amount of time physicians spend in pathway C, D and E with teledermoscopy can be estimated as displayed in Table 2.

## Discussion

### Principal Findings

The use of teledermoscopy by PCPs prolonged the diagnostic process, whereas the process was significantly shortened for dermatologists. The constructed pathway C (benign lesion) was shortened for physicians with teledermoscopy, while the diagnostic pathways A (benign lesion), B (NMSC), D (MM), and E (MM) were prolonged by 16%‐58%.

### Comparison With Previous Work

#### Primary Care Providers

Only a few studies have looked at the duration of consultations with teledermoscopy at PCPs. Similar to our findings, Berghout et al [17] and Nami et al [18] both reported prolonged consultation times compared to consultations without teledermoscopy. They found an average duration of consultations with teledermoscopy of 11:32 and 19:00, respectively [17,18]. This is longer than the median consultation time of 09:10 (IQR 08:19-11:59) in our study, but a direct comparison is challenging because of variations in the many components forming a typical consultation and the use of different types of teledermoscopy technologies.

Time stamps specifying how much time PCPs spent exclusively on teledermoscopy were not registered during time recordings in our study, yet the time can be estimated to be 05:09, based on the median time used for consultations with (09:10) and without (04:01) teledermoscopy. Nami et al [18] and van Sinderen et al [12] reported a mean and median time consumption solely for teledermoscopy usage of 04:00 and 05:24, respectively. Furthermore, based on the data provided by Berghout et al [17], it can be calculated that the time spent only on teledermoscopy was 06:49 (11:32 minus 04:43). Again, a statistical comparison of these results with our estimations is difficult due to variations in method, technical equipment, and the absence of statistical data, such as SD and range in some of the studies.

In the randomized controlled trial by Berghout et al [17], the authors discovered that in the first consultations, both the duration of the consultation and the task of filling out the telereferral were significantly prolonged compared to the later consultations. This tendency was also observed by van Sinderen et al [12]. During our data collection, 3 out of 5 PCPs included in the teledermoscopy group expressed a lack of confidence in using the teledermoscopy equipment because they had not used it for some time. This may have affected our results and prolonged some of the recordings. In 2 out of 6 of our time recordings with teledermoscopy, the PCPs had to get the teledermoscopy device from a colleague during the consultation, thereby extending the time recordings. This aspect however, might just reflect the current conditions in a real work setting where a number of PCPs in a primary care center share the teledermoscopy equipment.

Despite recordings with teledermoscopy being longer than those without, the duration was still within the time limit of 15 minutes that most PCPs allocate for consultations. On the other hand, it is not uncommon for patients to raise concerns about skin issues as additional topics during consultations, which may necessitate reducing the amount of time spent with teledermoscopy. In our study, all images were obtained by the PCPs themselves. Since the beginning of this project, teledermoscopy has been implemented at numerous primary care centers where the process of obtaining the photos has been assigned to other staff (eg, nurses), which could solve this issue.

#### Dermatologists

In contrast to the prolonged consultations at PCPs, teledermoscopy significantly shortened the examination of the skin lesions for dermatologists. Other studies reported that evaluation of telereferral by dermatologists lasted between 01:05 and 02:30 [12,18,19], which is consistent with our results. None of the studies compared their measurements with the duration of in-person consultations with a dermatologist.

#### Pathologists

Given that we only measured a part of the histopathological evaluation, in a number of cases, it is likely that the median time pathologists spend in our study is shorter than the time they typically spend examining these lesions. However, since we used the same calculated median for pathologists in all the constructed diagnostic pathways with and without teledermoscopy, it does not influence the absolute time difference in these pathways.

In Denmark lesions suspected of melanoma are analyzed within 7 days [26], while carcinomas and other skin biopsies may take longer. This aspect is not accounted for in the time recordings.

#### Constructed Diagnostic Pathways

Alternative diagnostic pathways could have been constructed, which might have changed the outcome. For instance, the simplification of the pathways could overlook larger time differences in more complicated patient courses. However, we argue that the displayed pathways in this study were the most common based on previous experience and the pathways described in the national guidelines.

According to our calculations, dermatologists, plastic surgeons, and pathologists could overall save time with teledermoscopy, while PCPs would spend more time using the teledermoscopy equipment. To provide perspective, PCPs constitute the largest specialty group in Denmark, including about 3500 physicians [27]. Consequently, each PCP would spend slightly more than 3 hours per year or less than 1 minute a day on average, using teledermoscopy. Enhancing PCPs’ skills might reduce the amount of time PCPs spends with teledermoscopy further.

In pathway B, the duration of the physical consultation at the dermatologist might be reduced, as the dermatologist had previously examined the lesion using teledermoscopy, and therefore, only needed to treat the lesion. Similarly, information regarding the size and placement of the skin lesion obtained with teledermoscopy could be beneficial in the planning of the surgical procedure at the department of plastic surgery, thereby making a physical preliminary examination of the skin lesion unnecessary in pathways D and E.

It is important to notice, that the focus of this study was solely on physicians “hands-on” time consumption. Hence, additional time required for tasks, such as preparation time and tissue slide collection, was not included. Furthermore, because most diagnostic pathways involve secretaries, nurses, and medical laboratory technicians, preventing benign skin lesions from unnecessary treatment might save time for other medical staff as well.

Patient resources are also crucial when considering the implementation of teledermoscopy. While teledermoscopy might save time for specific groups of physicians, we did not consider the impact on patients time or the overall duration of the diagnostic process for patients. Fortunately, other studies have looked into this. A review by Jones and Oakley [11] found that the majority of studies investigating time outcomes for teledermatology reported reduced time from referrals to biopsy and treatment with teledermatology. The study by van Sinderen et al [12] reviewing 11 years of teledermoscopy in the Netherlands, found that the median response time for teledermatologists was 2.4 hours compared to an average waiting time of 2.8 weeks for in-person consultations. Furthermore, several studies reported that the number of face-to-face consultations with dermatologists was reduced with teledermoscopy [12,28,29]. Consequently, fewer patients and accompanying persons might have to take time off from work to attend in-person consultations [8].

### Strengths and Limitations

This study has several limitations. First, due to limited resources and different geographic locations, it was not possible to follow each patient throughout the diagnostic process. This resulted in fragmented measurements of physician’s time consumption, involving independent groups of patients. Second, identifying relevant cases was difficult, especially at the PCPs, because patients have a tendency to bring up skin problems as supplementary subjects during consultations. Consequently, we were unable to achieve the intended number of patients and most time recordings were performed on patients with benign skin tumors. Third, our data are very heterogeneous due to the nature of the different departments included and the large spectrum of potential diagnoses. Fourth, patients with multiple potential skin cancers were not included in this study. Finally, this study was mainly based on structures in the Danish health care system. Some of our results might not apply directly to other health care systems.

This is one of few studies measuring clinicians’ time, as suggested by a recent review [30]. A strength of the study is that all time recordings were obtained in a real work setting, which gives the results high external validity. Furthermore, the same person performed the majority of the recordings.

### Further Research

More time recordings would be required to obtain a more accurate picture of physicians’ time consumption, for example, by including more departments and primary care centers, increasing the number of data collectors, or involving medical staff at the departments to a greater extent. In addition, it would increase data transparency if time stamps stating how much time PCPs and pathologists spend on each task were registered during the recordings. Altogether, several modifications to this pilot study’s design would be required to make it feasible for a larger-scale investigation.

### Conclusion

Teledermoscopy significantly shortened the diagnostic process for dermatologists but prolonged it for PCPs. Previous studies have concluded that teledermoscopy reduces the number of physical referrals as well as surgical procedures, and our time study indicates that this would altogether result in time saving for dermatologists, plastic surgeons, and pathologists. Part of the time savings attained might be reallocated to other areas of the health care system, such as consultations at PCPs.

## Abbreviations

CPP: cancer patient pathway
MM: malignant melanoma
NMSC: nonmelanoma skin cancer
OUH: Odense University Hospital
PCP: primary care provider
